# GenoFig: a user-friendly application for the visualization and comparison of genomic regions

**DOI:** 10.1093/bioinformatics/btae372

**Published:** 2024-06-13

**Authors:** Maxime Branger, Sébastien O Leclercq

**Affiliations:** INRAE, Université de Tours, ISP, Nouzilly F-37380, France; INRAE, Université de Tours, ISP, Nouzilly F-37380, France

## Abstract

**Motivation:**

Understanding the molecular evolutionary history of organisms usually requires visual comparison of genomic regions from related species or strains. Although several applications already exist to achieve this task, they are either too old, too limited, or too complex for most user’s needs.

**Results:**

GenoFig is a graphical application for the visualization of prokaryotic genomic regions, intended to be as easy to use as possible and flexible enough to adapt to a variety of needs. GenoFig allows the personalized representation of annotations extracted from GenBank files in a consistent way across sequences, using regular expressions. It also provides several unique options to optimize the display of homologous regions between sequences, as well as other more classical features such as sequence GC percent or GC-skew representations. In summary, GenoFig is a simple, free, and highly configurable tool to explore the evolution of specific genomic regions in prokaryotes and to produce publication-ready figures.

**Availability and implementation:**

Genofig is fully available at https://forgemia.inra.fr/public-pgba/genofig under a GPL 3.0 license.

## 1 Introduction

Comparative genomics is the method of choice to investigate adaptation through genetic rearrangement or horizontal gene transfer, since these events create changes in gene synteny between related organisms. The field greatly benefited from the generalization of next generation sequencing in the last 15 years, resulting in the publication of hundreds of thousands of new genomes representing thousands of living species. The recent development of long-read sequencing now provides assembly of complete genomes (Todd *et al.* 2018, Nurk *et al.* 2022), including complex regions usually associated with repeated elements. This is particularly meaningful in prokaryotes, where plasmids and other transferable bacterial mobile genetic elements (MGEs) are made up of several functional blocks exchangeable between elements and often carry highly variable accessory regions (Toussaint and Merlin 2002, Partridge *et al.* 2018).

A popular way to understand the evolution of a locus of interest is to produce a graphical representation of its genomic context in various genomes, helping visualizing changes of synteny, gene content, or breaks in nucleotide composition or divergence. One of the first and most widely used tool dedicated to this purpose in prokaryotes is Easyfig (Sullivan *et al.* 2011), but the program has not been updated since 2020, is developed in the deprecated Python version 2.7, and has a low level of customization not fit for the current needs in comparative genomics. A series of other tools were subsequently developed to look at the gene content of a genomic region in several bacterial genomes simultaneously (Martinez-Guerrero et al. 2008, Garcia *et al.* 2019, Gumerov and Zhulin 2020, Saha *et al.* 2021, Botas *et al.* 2022). However, none of them have an option to show homologous regions or nucleotide composition, which are essential features when studying genomic evolution. Moreover, they are usually designed to focus on a specific gene and display a fixed number of upstream and downstream genes, often from genomes stored in a pre-defined database. In that, they are not suitable to display the several dozens of Kbps of complete MGEs or to compare unpublished sequences. Softwares specifically designed for the comparison of large prokaryotic genomic regions are available but suffer from several other limitations. BRIG (Alikhan *et al.* 2011) allows the comparison of whole plasmid sequences in a circular display, but is based solely on the presence/absence of genes compared to a reference genome, without considering synteny. On the opposite, Mauve (Darling *et al.* 2010) was designed to display synteny breaks between large sequences using-color coded synteny blocks, but its representation of genetic annotations is very limited and is best fitted to understand genetic rearrangements at the whole genome level. GenomeMatcher (Ohtsubo *et al.* 2008) is another tool implemented for the comparison of large genomic regions, which provides a full set of modules including parallel and dot plot view, as well as synteny breakpoint analysis. However, it is limited to the comparison of only two sequences, making it not suitable for most comparative genomics analysis.

Another aspect which has to be kept in mind when developing a genomic visualization tool is its ease of use and its level of configuration. Current annotated sequence formats, such as GenBank or EMBL formats, provide information for several types of genomic regions, referred as features. Features may represent genes and coding regions (CDS), but also more specific regions such as ribosomal and transfer RNA, repeated regions, or mobile elements. All these features have their own information such as gene name, function of the encoded protein, or the kind of tRNA. An efficient visualization tool should allow the representation of these features in various styles depending on their annotation, with minimal effort. Some tools, such as GenomeDiagram (Pritchard *et al.* 2006), Gggenomes (Hackl and Ankenbrand 2024), Clinker (Gilchrist and Chooi 2021), or Gene Graphics (Harrison *et al.* 2018) have been designed to provide user-defined representation of annotated features for several sequences, but they require either extensive programming knowledge or to manually set up configuration for each feature of each sequence individually.

With all these considerations in mind, we developed GenoFig, a tool for the visual comparison of prokaryotic genome regions originally based on the drawing engine of Easyfig 2 but with a brand new interface and several new implementations, generating highly personalized publication-quality figures.

## 2 Application description

### 2.1 User-friendly interface

GenoFig was primarily designed to be as user-friendly as possible, with the possibility to generate publication-level figures within a few clicks. It is composed of a unique window divided in panels, one for each component type of the figure: sequences, features, homologies, legends, and additional decorations. Components in the sequence, feature, and decoration panels are organized in lists with an unlimited number of entries, and with all parameters directly modifiable with a single mouse click. Generating a figure usually proceeds in the following steps: load sequence(s), configure their drawing style, define features to draw and their style, run BLAST search between sequences if homology is expected, configure legends and scale, then create the figure in SVG or PNG format. In the resulting figure, sequences are all drawn one below each other in the order of the sequence panel. All sequences and features are drawn on scale, defined by the size of the largest displayed sequence. GenoFig can read sequences in either GenBank or FASTA formats. GenBank-formatted files are preferred to FASTA-formatted files since they contain annotated features in addition to the nucleotide sequence. They can be for instance downloaded from the NCBI Nucleotide website under the .gb file extension (using the “send to > Complete Record > File > GenBank (full)” link on an accession page) or provided by most annotation software under the .gbk or .gbff file extensions. Multiple homologous or unrelated sequences included in the same file are loaded as independent sequence entries. Loaded sequences can be easily reorganized through drag-and-drop functionality, removed, or simply inactivated. This last option allows the user to hide sequences without removing them, therefore keeping all their configuration.

**Figure 1.**
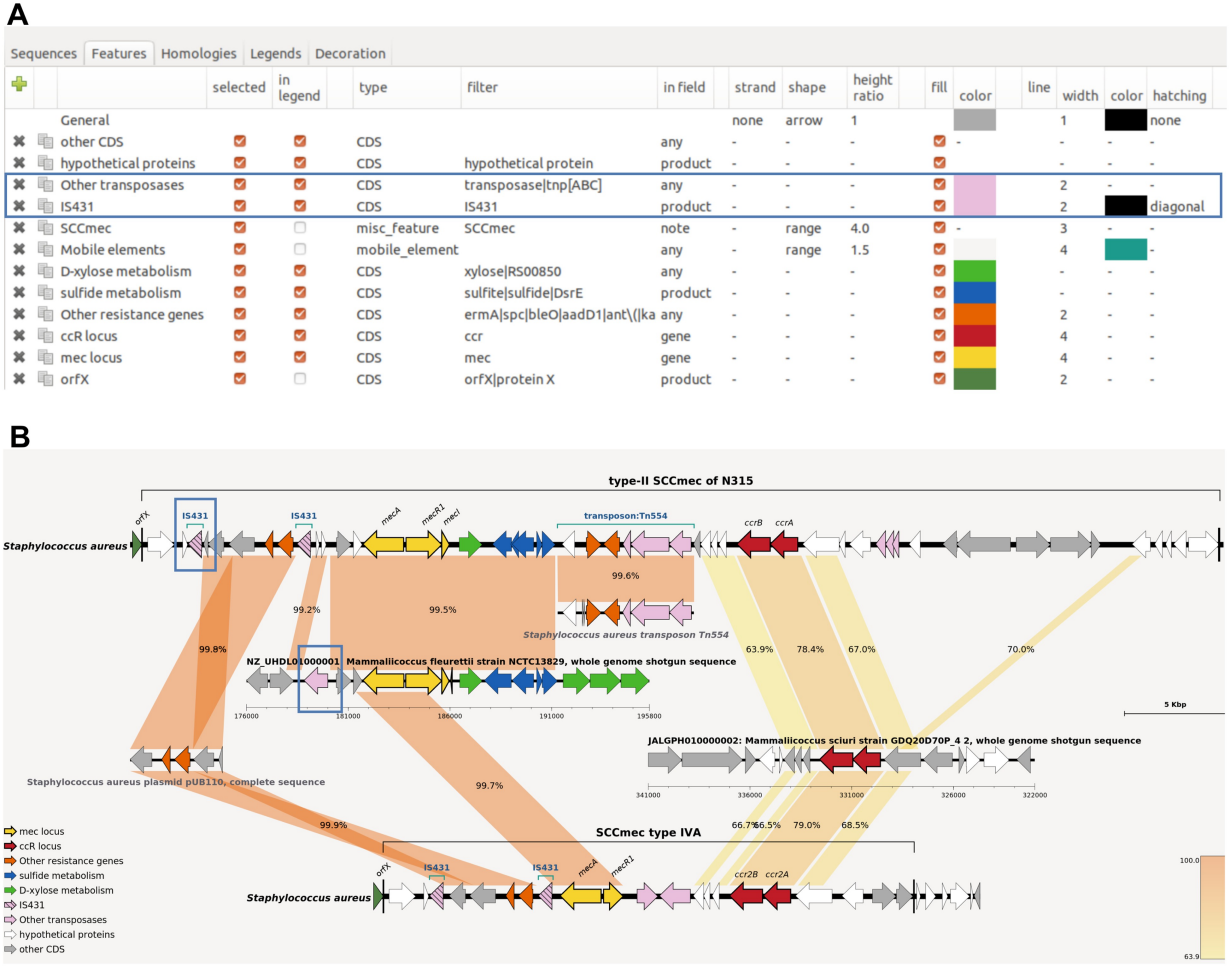
Features panel of GenoFig (**A**) and the corresponding generated figure (**B**). Options for drawing generic and IS*431* transposases in (A) and the resulting drawing in (B) are boxed in blue. The “custom blast” option of GenoFig was used to specifically display homologies between the two SCC*mec* elements and other genomic sequences, highlighting the modular origin of these methicillin resistance elements. Annotated sequences of mobile elements SCC*mec* type II, type IVA, Tn*554* transposon, and plasmid pUB110 were downloaded from GenBank (accession numbers D86934, EU437549, X03216, and NC_001384, respectively). Genomic regions for *Mammaliicoccus fleurettii* NCTC13829 and *Mammaliicoccus sciuri* GDQ20D70P_4 were downloaded from RefSeq and GenBank, respectively; their accession numbers are provided in the figure.

One of the major needs in comparative genomics is to be able to draw different loci with specific color or shape depending on their function or other attribute of interest. In GenoFig, annotated features can be drawn in a variety of styles defined by the user (Fig. 1A). Global specifications can be applied to each feature type (CDS, tRNA, mobile element, …), but a key component of GenoFig is to propose feature-specific configurations using word-matching queries. Using the filter parameter, one can choose to apply a set of properties (color, shape, size, hatching, …) to features depending on regular expressions found in the gene name, product, locus tag, or even the notes. This filter will apply to all sequences, providing a consistent representation of annotated features thorough the figure. For instance, CDS encoding transposases can be colored in pink in all sequences by creating a new feature specification using the filter “transposase|tnp[ABC]” in field “any” with a fill color to “pink” (Fig. 1A). This filter is constructed as a regular expression meaning “transposase or tnpA or tnpB or tnpC” and will be searched in all fields of annotated CDS, allowing the detection of transposases by their product (“transposase of…”) or by their gene name (“tnpA/B/C”). Moreover, feature specifications can be overridden by more specific ones, to better highlight genes or regions of interest. Following the same example, adding another feature specification using the filter “IS431” in field “product” with the hatching option to “diagonal” will highlight only transposases of the IS431 family with diagonal hatching while retaining other transposases as pink with no hatching (Fig. 1). The number of feature specification is not limited, allowing a very fine-tuned representation of annotated sequences.

### 2.2 High level of configuration

Although GenoFig allows the production of figures very quickly, its main interest lies on the high level of configuration achievable. In version 1.0, 15 configuration parameters are available for sequences drawing and 19 for features drawing. Most of these parameters can be configured for a single feature, globally for all features, or can be sequence-specific. This modularity allows the production of complex figures in a rather simple way. As stated in Section 1, being able to accurately represent regions of homologies between sequences is one of the most important implementation required when doing comparative genomics, to be able to understand complex structural changes produced by rearrangements. The homologies panel of GenoFig allows a fully customizable representation of homologous regions detected by BLASTN or TBLASTX programs (Camacho *et al.* 2009).

BLASTN is run with default parameters and TBLASTX is run using the bacterial genetic code and a minimal e-value of 10^−3^. BLAST searches are performed only once between all sequences included in the sequence panel, and the results are saved in a file. Blast hits can then be filtered by size, similarity threshold, or e-value using the interface. To fully understand the evolution of complex genomic regions, it is often necessary to look at shared homologies between several sequences, and not only between adjacent sequences in the drawing. The “custom blast” option of GenoFig was specifically designed for this purpose, allowing the user to precisely choose between which sequences to draw homologies. Figure 1B represents two variants of the *Staphylococcus aureus* SCC*mec* element, a typical prokaryotic mobile element made up of several unrelated genetic regions. According to the current theory (Tsubakishita *et al.* 2010, Lakhundi and Zhang 2018), most SCC*mec* types originated from an integration of the chromosomal methicillin resistance gene *mecA* of *Mammaliicoccus fleurettii* and its regulatory locus in a *Mammaliicoccus* SCC element (encoding Ccr2A and Ccr2B recombinases) already circulating among *Staphylococcal* species. Additional mobile elements carrying other antibiotic resistance genes, such as pUB110 or Tn554, could also be integrated depending on the SCC*mec* type. To our knowledge, no other tool could summarize on a single figure the complex organization of this element without relying on programming skills or extensive manual drawing/modifications.

### 2.3 Other implementations

An interesting implementation in GenoFig is the possibility of automatic sequence positioning. Using the “best blast” sequence option, horizontal coordinates of sequences in the figure are computed in a way that the largest shared region between two sequences is on the same vertical axis. This parameter helps to highlight regions of homology when many sequences are displayed on a figure. It can also be used to efficiently map contigs of a draft genome on a reference in order to quickly determine completeness and potential rearrangements (Supplementary Fig. S1). Finally, some complementary information related to the DNA sequence itself, such as GC content, GC-skew, or a sequence-specific scale, can be printed using the decoration panel. These additional information are again fully configurable, and can be drawn for all sequences or for a subset of sequences only (Supplementary Fig. S1).

All parameters modified to produce a figure can be saved in a project file, which can later be loaded to recreate the same figure or modify it. GenoFig also offers the possibility to save display parameters into configuration files. These files contain only feature, homology, legend, and decoration parameters. This option allows the user to reuse the exact same parameters on several sets of sequences, to provide a consistent display across generated figures without the need of configuring everything manually every time.

### 2.4 Technical implementation

GenoFig is a stand-alone Python3 application with a GTK3-encoded graphical user interface. The drawing engine of GenoFig is based on the open-source code of Easyfig 2 (https://github.com/mjsull/Easyfig), with some major reimplementation. Parsing of GenBank and Blast files are performed using various modules from the Biopython package (Cock *et al.* 2009). The module svg2png from the cairosvg package is used to convert output images from SVG to PNG format. GenoFig can be executed on Linux, MacOS, and Windows platforms in a conda environment containing all required python packages. For users not familiar with command-line and conda environments, single-file executable versions were compiled for Windows and MacOS platforms. These release versions may however not include all future improvements or bug fixes.

## 3 Conclusion

GenoFig has been designed to be both simple and complete, with the intent to be useful for exploring genetic evolution hypotheses as well as for producing publication-ready figures. Although many options were considered, this first released version still has room for improvements. For instance, GenoFig heavily relies on the annotation quality of input files, which may be inconsistent between sequences annotated manually or using different pipelines. Future implementation may include automatic spread of annotations from a well-defined reference to other sequences according to user-specified homology thresholds. Moreover, GenoFig’s main purpose is to display prokaryotic regions of up to several hundred of Kbp, and is not intended to compare multiple bacterial chromosomes. Future versions of the application could include the open-source progressive Mauve algorithm (Darling *et al.* 2010) in addition to BLAST searches to cope with complete bacterial genome comparisons, or include the recognition of eukaryote-specific features such as intron/exon delimitations to enable the comparison of small eukaryotic genetic regions. Finally, GenoFig is an open-source software, which means that users can develop and share their own modules to answer some of their favorite questions. In that, we hope that GenoFig could become an ever-improving tool meeting the needs of anyone interested in evolutionary genomic questions.

## Supplementary Material

btae372_Supplementary_Data

## Data Availability

All data underlying this article are available in GenBank and RefSeq Nucleotide Databases under accession numbers provided in the figure legends.
