# Supplementary material for: GenoFig: a user-friendly application for the visualization and comparison of genomic regions
Source: Bioinformatics. 2024 Jun 13;40(6):btae372. doi: 10.1093/bioinformatics/btae372 (PMC11199195; doi:10.1093/bioinformatics/btae372)
Supplement: btae372_Supplementary_Data [file btae372_supplementary_data.zip › Bioinformatics_Suppl_Figure1_REVISED.v2.pdf]

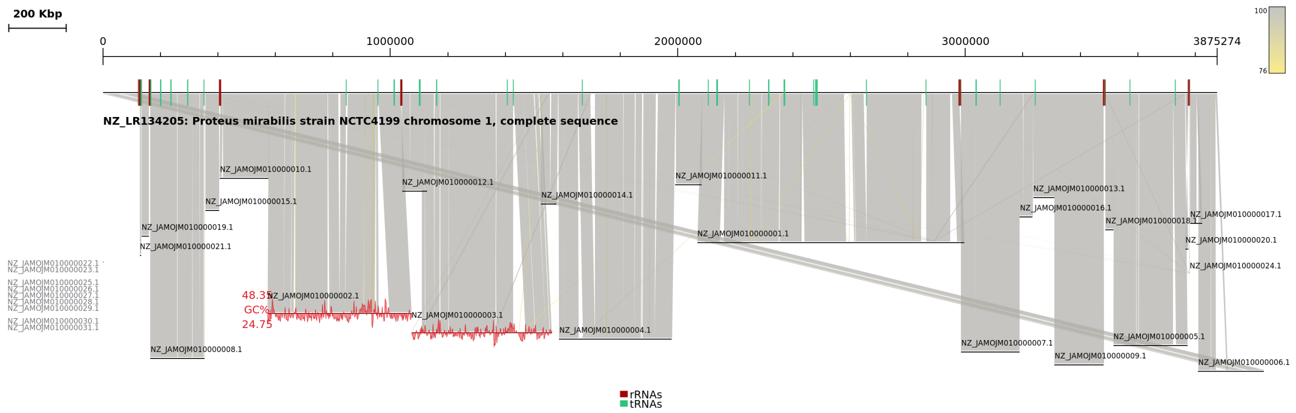

Supplementary Figure S1

Alignment of complete and draft *Proteus mirabilis* assemblies, showing draft genome completeness and insertions of large genomic regions adjacent to several tRNAs. The complete genome of *P. mirabilis* NCTC4199 was downloaded from RefSeq (Accession NZ_LR134205) in GenBank format and the draft genome of *P. mirabilis* FZP3320 was downloaded in multi-fasta format from the Assembly database (Accession GCF_025379525, contig names NZ_JAMOJM). Contigs were drawn using an in-between sequence space of 10 px and the ‘best-blast’ option, to position them according to their best matching region in the reference. A few contigs (in gray at the leftmost of the image) were not mapped, either because they were smaller than the 1000 bp threshold we set up for homology display or because they were absent from the reference. Annotated rRNA and tRNA as well as the scale were drawn on the reference sequence, and GC content was displayed in red for two contigs. The final figure was prepared in less than 20 minutes.
